# DNA methyltransferase 3 beta regulates promoter methylation of microRNA-149 to augment esophageal squamous cell carcinoma development through the ring finger protein 2/Wnt/β-catenin axis

**DOI:** 10.1080/21655979.2022.2031411

**Published:** 2022-02-06

**Authors:** Junfeng Yang, Quan Zhang, Pu Zhao, Tong Qiao, Zhikun Cao, Fei Gao, Mengbo Liu, Sen Wu

**Affiliations:** Department of Thoracic Surgery, Zhengzhou Key Laboratory of Surgical Treatment for End-stage Lung Diseases, Henan Provincial People’s Hospital, People’s Hospital of Zhengzhou University, Zhengzhou, P.R. China

**Keywords:** Esophageal squamous cell carcinoma, *DNMT3B*, methylation, *miR-149*, *RNF2*, wnt/β-catenin

## Abstract

Esophageal squamous cell carcinoma (ESCC) is an aggressive form of human squamous cell carcinomas with extremely aggressive pathological features. This study explores the functions of microRNA-149 (*miR-149*) and its interacted molecules in ESCC. The ESCC-related miRNA and messenger RNA (mRNA) datasets were applied to identify aberrantly expressed genes in ESCC. Forty-two patients with ESCC were included and their tissue samples were collected. *miR-149* was poorly expressed whereas DNA methyltransferase 3 beta (*DNMT3B*) and ring finger protein 2 (*RNF2*) were abundantly expressed in ESCC tumor samples. Overexpression of *miR-149* suppressed growth and invasiveness of ESCC cells *in vitro* and *in vivo. DNMT3B* bound to the promoter region of *miR-149* to trigger its promoter methylation and downregulation. *RNF2* mRNA was a target of *miR-149. RNF2* overexpression blocked the inhibitory effect of *miR-149* on ESCC cell growth. *RNF2* activated the Wnt/β-catenin pathway to promote ESCC development. In conclusion, this study found that *DNMT3B* downregulates *miR-149* level through methylation modification of the *miR-149* promoter, while *miR-149* suppresses *RNF2* expression and inactivates the Wnt/β-catenin pathway to suppress growth of ESCC cells.

## GRAPHICAL ABSTRACT

**Figure:**
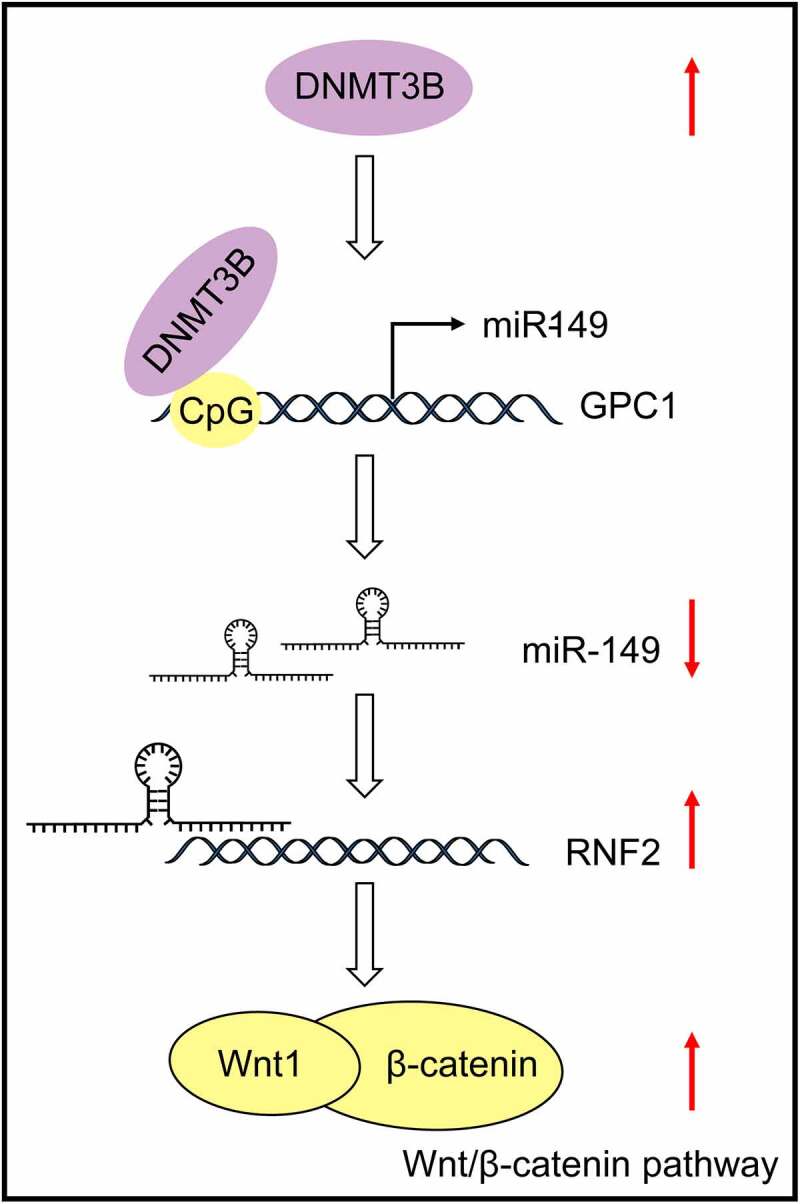

## Introduction

Esophageal carcinoma (ESCA) is a malignant digestive tract cancer that is categorized into esophageal squamous cell cancer (ESCC) and esophageal adenocarcinoma [1]. The former type is more frequent in developing nations, including China, whereas the latter type is more common in western countries [2]. Current treatments of ESCC include surgical resection, interventional therapy, chemotherapy, radiotherapy, and targeted therapy [3]. Surgery is an effective option for the management of early cancer, but patients are often diagnosed at advanced stages that are no longer candidates for surgery, whose 5-year survival rate was as low as 15%–25% [4]. As a deadly disease, ESCC requires extensive research. An early diagnosis is crucial for ESCC control, but the development of nonsurgical therapy is also of great importance, which requires more understandings of the molecular mechanisms involved in the disease development.

MicroRNAs (miRNAs) are a subclass of non-coding RNAs that participate in an array of biological processes, and dysregulation of miRNAs is commonly found in almost every tumorigenic process [5]. The versatile functions of miRNAs are attributed to their potent regulation on target mRNAs through binding to the 3ʹUTR [6]. miRNAs are also suggested as potential biomarkers and therapeutic options for ESCC as well [7]. In this research, by using bioinformatics tools, we found that *miR-149* was abnormally poorly expressed in ESCC samples. *miR-149* has reportedly been dysregulated in many human cancers, playing either oncogenic or anti-cancer roles depending on the specific cancer types [8]. In ESCC, downregulation of *miR-149* by circular RNA 0000654 (*hsa_circ_0000654*) triggered the malignant behaviors of ESCC cells [9]. In addition, our integrated bioinformatics analyses confirmed ring finger protein 2 (*RNF2*) as a target transcript of *miR-149*. High *RNF2* expression was detected in tissues of ESCC patients and linked to increased tumor volume [10]. We therefore wondered that poor expression of *miR-149* expression in ESCC might be relevant to ESCC development. Upregulation of *miR-149* would possibly upregulate *RNF2* to suppress ESCC development.

DNA methylation, which usually occurs at the CpG site, has critical biological roles including gene expression regulation and chromatin structure, and methylation within the CpG Islands influences multiple cellular functions [11]. In this research, we observed that the CpG Island of the *miR-149* promoter was highly methylated. Three catalytic active DNA methyltransferases (DNMTs), *DNMT1, DNMT3A*, and *DNMT3B*, have been confirmed in mammals [12], and abnormal activity or polymorphism of these DNMTs is related to human diseases [13]. *DNMT3B* is the main DNMT activated during embryonic development, and it also functions as the major enzyme methylating intragenic regions of active genes and is linked to the onset and progression of specific pathologies [14]. By using bioinformatics tools, we predicted direct-binding site between *DNMT3B* and the promoter region of *miR-149*. Taken together, we hypothesized that *DNMT3B* possibly mediates *miR-149* promoter methylation to induce its downregulation and *RNF2* restoration, which therefore leads to ESCC progression. This study aimed to validate the interactions between *DNMT3B, miR-149, and RNF2* and their involvements in the ESCC cellular behavior alterations *in vitro* and *in vivo*.

## Materials and methods

### Bioinformatics analyses

Three ESCC-related datasets, including a miRNA dataset GSE67268, and two mRNA datasets GSE29001 and GSE20347, were obtained from Gene Expression Omnibus (GEO; https://www.ncbi.nlm.nih.gov/geo) to identify differentially expressed (DE) genes. All data were analyzed utilizing the Affymetrix R Package (http://www.bioconductor.org/packages/release/bioc/html/affy.html). The DE genes were screened using an R limma Package (http://master.bioconductor.org/packages/release/bioc/html/limma.html). The criteria for gene screening were |Log FoldChange| > 1.5 and adjust *p* value < 0.05. The heatmaps were produced by an R pheatmap package (https://cran.r-project.org/web/packages/pheatmap). The potential target mRNAs of *miR-149* were predicted using three bioinformatic websites including StarBase (http://starbase.sysu.edu.cn/index.php), TargetScan (http://www.targetscan.org/vert_71/) and mirRDB (http://ophid.utoronto.ca/mirDIP/). In addition, the intersections of the predicted mRNAs, including those screened from the GSE29001 and GSE20347 datasets, were analyzed using the Jvenn system (http://jvenn.toulouse.inra.fr/app/example.html). DE genes in ESCA were further predicted in The Cancer Genome Atlas (TCGA)-ESCA database in the Gene Expression Profiling Interactive Analysis system (http://gepia.cancer-pku.cn/index.html).

### Clinical sample collection

From May 2017 to October 2018, 42 patients with primary ESCC treated at the People’s Hospital of Zhengzhou University were recruited into this research. This research was ratified by the Clinical Ethical Committee of the People’s Hospital of Zhengzhou University (Approval No. REC-HNPPH.20170513) and abided by *Helsinki Declaration*. Informed consent forms were received from all subjects. The average age of respondents was 61.37 years, ranging from 41 to 73 years. Among them, 19 were diagnosed at stage I ESCC, 16 at Stage II, and the rest 7 at Stage III–IV. All included patients were diagnosed as ESCC by imaging examination and pathologic biopsy. The clinical stages were confirmed by Computed Tomography and the metastasis was determined by ultrasonic examination. Patients with any other primary disease, or with a history of immune deficiency disease or infectious disease, or pregnant or lactating women were excluded. All included patients underwent surgical resection before radiotherapy. ESCC tumor and the para-tumorous healthy tissues were harvested during surgery. All the tissue samples were stored at −80°C. None of them received chemo- or radio-therapy before surgery.

### Cell treatment

A human esophageal epithelial cell line HET-1A (CC-Y1220) and an ESCC cell line EC9706 (CC-Y1571) were procured from EK Biosciences (Shanghai, China), another two ESCC cell lines TE1 (CL-0231) and KYSE150 (CL-0638), and the human embryonic kidney (HEK) 293 T cells (CL-0005) were procured from Procell Life Science & Technology Co., Ltd. (Wuhan, Hubei, China). Cells were cultured in 10% fetal bovine serum (FBS)-supplemented Dulbecco’s modified Eagle’s medium (DMEM; Gibco Company, NY, USA) at 37°C with 5% CO_2_. The sequences of *miR-149* and *RNF2* were inserted into pcDNA-3.1 plasmids (Thermo Fisher Scientific, Wilmington, DE, USA). The overexpression plasmids (miR-149 mimic and oe-RNF2) were transfected into cells according to the protocol of Lipofectamine 2,000 Reagent (Thermo Fisher Scientific). After 48 h, stably transfected were screened and collected for further use.

### Reverse transcription quantitative polymerase chain reaction (RT-qPCR)

Gene expression was detected by RT-qPCR. In brief, the RNA sample was extracted with an RNAiso Plus Kit (Takara Holdings Inc., Kyoto, Japan). The quality of extracted RNA was examined by the Nanodrop-2000C Kit (Thermo Fisher Scientific). After that, the RNA sample (500 ng) was reverse-transcribed to complementary DNA (cDNA) using a PrimerScript RT kit (Takara Biotechnology Ltd., Dalian, China). The RT system (20 μL) included 4 μL PrimeScript buffer, 1 μL Enzyme Mix I, 1 μL Oligo dT Primer, and 1 μL Random 6 mers, and the RT conditions were 37°C for 15 min, 85°C for 5s, and maintained at 4°C to obtain cDNA. Thereafter, qPCR was conducted with ChamQ SYBR Color qPCR Master Mix (Vazyme Biotech, Nanjing, Jiangsu, China) and a CFX96 PCR Kit (Bio-Rad, Hercules, CA, USA). The PCR system (20 μL) consisted of 10 μL ChamQ SYBR Color qPCR Master Mix, 0.4 µL forward primer, and 0.4 µL reverse primer. The cycling condition was: pre-denaturation at 95°C for 30s, 40 cycles of denaturation at 95°C for 10s and annealing at 60°C for 30s, and a final extension at 60°C for 60s. The primer sequences are presented in Table 1. Glyceraldehyde-3-phosphate dehydrogenase (GAPDH) and U6 small nuclear RNA were used as endogenous loadings. Fold change of gene expression was examined by the 2^−ΔΔCq^ method.

**Table 1.** Primer sequences for RT-qPCR

Gene	Primer sequence (5’-3’)
*miR-149*	F: TCTGGCTCCGTGTCTTC
	R: GAACATGTCTGCGTATCTC
*U6*	F: CTCGCTTCGGCAGCACAT
	R: TTTGCGTGTCATCCTTGCG
*DNMT1*	F: AGGTGGAGAGTTATGACGAGGC
	R: GGTAGAATGCCTGATGGTCTGC
*DNMT3A*	F: CCTCTTCGTTGGAGGAATGTGC
	R: GTTTCCGCACATGAGCACCTCA
*DNMT3B*	F: TAACAACGGCAAAGACCGAGGG
	R: TCCTGCCACAAGACAAACAGCC
*DNMT3L*	F: GTTCGTGGACAATCTGGTGCTG
	R: CGGACAGCATTCTGCAAGGATC
*E-cadherin*	F: GCCTCCTGAAAAGAGAGTGGAAG
	R: TGGCAGTGTCTCTCCAAATCCG
*ZO-1*	F: GTCCAGAATCTCGGAAAAGTGCC
	R: CTTTCAGCGCACCATACCAACC
*N-cadherin*	F: CCTCCAGAGTTTACTGCCATGAC
	R: GTAGGATCTCCGCCACTGATTC
*Vimentin*	F: AGGCAAAGCAGGAGTCCACTGA
	R: ATCTGGCGTTCCAGGGACTCAT
*RNF2*	F: CGCATCAGGAAAGGGTCTTAGC
	R: CTATCTGCTGCTTTTTGCCTCGC
*GAPDH*	F: GTCTCCTCTGACTTCAACAGCG
	R: ACCACCCTGTTGCTGTAGCCAA

RT-qPCR, reverse transcription quantitative polymerase chain reaction; miR-149, microRNA-149; DNMT, DNA methyltransferase; ZO-1, tight junction protein 1; RNF2, ring finger protein 2, GAPDH, glyceraldehyde-3-phosphate dehydrogenase; F: forward; R, reverse.

### 5-ethynyl-2’-deoxyuridine (EdU) labeling assay

The transfected cells were cultured in 96-well plates in triplicate (4 × 10^4^ cells per well). After 48 h, each well was loaded with 100 μL EdU reagent (Solarbio Science & Technology, Beijing, China) for 2 h. Thereafter, cells were further incubated with cell fixing reagent (100 μL per well), 2 mg/mL glycine, and penetrant (100 μL per well, 0.5% Triton X-100-supplemented phosphate-buffered saline at 22–25°C for 30 min. After that, cells were stained with 1× Apollo and 1 × Hoechst 33,342 solutions (100 μL per well), and then incubated with the anti-fluorescence quenching solution (100 μL per well). The labeling was observed under a microscope with three random fields included. The EdU-positive cells were stained in red while total cells were in blue. The EdU-positive rate (%) was calculated as follow: rate = number of EdU-positive cells/number of total cells × 100% [15].

### Colony formation assay

A total of 1 × 10^3^ transfected cells were cultured in a 90-mm dish containing 5 mL complete 10% FBS-DMEM in a 37°C sterile incubator for 2 weeks. The medium was renewed every 2 days. Thereafter, the cells were washed, fixed, and stained with 0.1% crystal violet at 22–25°C for 10 min. The cell colonies (>50 cells) were counted under the microscope [16].

### Caspase-3 activity measurement

Transfected KYSE-150 or TE-3 cells were sorted in 96-well plates in triplicate (5 × 10^3^ cells per well). Each well was loaded with 100 μL Caspase-Glo 3/7 reagent and rotated on a shaking table for 2 min. Later, cells were incubated at 20°C for 3 min, and the fluorescence activity of each well at the excitation wavelength of 485 nm and the emission wavelength of 527 nm was examined using the microplate reader (PerkinElmer, Waltham, MA, USA).

### Flow cytometry

An Annexin V-fluorescein isothiocyanate (FITC)/propidium iodide (PI) kit (BD Biosciences, NJ, USA) was used to examine cell apoptosis. Briefly, transfected KYSE-150 and TE-1 cells were seeded in 6-well plates. The cells were suspended in binding buffer and labeled with Annexin V-FITC and PI for 15 min in a dark environment. The labeled cells were analyzed on the flow cytometer (BD Biosciences). The apoptosis rate of cells was examined by Flow Jo program (NIH, Bethesda, MD, USA) [17].

### Transwell assays

Transfected KYSE-150 and TE-1 cells were harvested and resuspended in serum-free DMEM to 1 × 10^5^/mL. Thereafter, 200 µL suspension was added to the apical chambers, which were precoated with Matrigel. The apical chambers were inserted into 24-well plates, while the basolateral cells were loaded with 500 µL 10% FBS-contained DMEM. The cells were cultured at 37°C for 24 h. After that, the membranes were collected, and the invasive cells were collected, fixed, and stained with crystal violet for 10–15 min. The invasive cells were observed under the microscope at a × 200 magnification. Cell migration was examined in a similar way except for the Matrigel pre-coating on apical chambers [18].

### *Induction of xenograft tumors* in vivo

Twenty-four NOD/SCID nude mice (4 weeks old, 20 ± 2 g) were procured from SLAC Laboratory Animal Co., Ltd. (Shanghai, China). Transfected KYSE-150 or TE-1 cells (7 × 10^6^ cells/mL) were transplanted into mice by subcutaneous injection. After that, the growth of xenograft tumors in mice was detected once per week for five weeks. Thereafter, the mice were sacrificed via 120 mg/kg pentobarbital sodium (intraperitoneal injection). The xenograft tumors were weighed and preserved at −80°C for subsequent histological examination. All animal procedures were authorized by the Animal Ethics Committee of the People’s Hospital of Zhengzhou University (Approval No. Z20190308G) and adhered to the Guidelines for Animal Care and Use (NIH, Bethesda, Maryland, USA) [19].

### Immunohistochemistry (IHC)

The harvested xenograft tumor tissues were made into 4-μm serial slices. The slices were dewaxed, rehydrated, and soaked in 3% H_2_O_2_ for 10 min. Next, the slices were hybridized with anti-KI67 (1:200, ab15580, Abcam Inc., Cambridge, MA, USA) , anti-DNMT3B (1:100, sc-376043, Santa Cruz, CA, USA), anti-RNF2 (1:100, sc-101109, Santa Cruz) and anti-proliferating cell nuclear antigen (PCNA; 1: 200, #13,110, Cell Signaling Technology (CST), Beverly, MA, USA) overnight at 4°C, and then with horseradish peroxidase (HRP)-labeled immunoglobulin G (IgG; #7076, CST) at 20°C for 1 h. Thereafter, the slices were counter-stained with hematoxylin for 20 s, and then sealed and observed under the microscope [20].

### Terminal deoxynucleotidyl transferase (TdT)-mediated dUTP nick end labeling (TUNEL)

Cell apoptosis in tissues was determined by the TUNEL assay. The tumor slices were stained by DAPI (1:5,000, Beyotime Biotechnology, Shanghai, China) for 30 min, and then stained using a TUNEL kit (Roche Ltd., Basel, Switzerland) according to the kit’s instructions. The TUNEL-positive cells were counted under the microscope (SP8, Leica, Solms, Germany) to evaluate the apoptosis activity [21].

### Methylation-specific PCR (MSP)-qPCR

Human genome DNA from tissues and cells was obtained using a genome DNA kit (TIANGEN, Biotech Co., Ltd., Beijing, China. The DNA was modified by sodium bisulfite using a ND methylation kit (Zymo Research, Orange, CA, USA) following the manufacturer’s protocol. The methylation and demethylation primers for MSP were synthesized by Sangon Biotech (Shanghai, China). Modified DNA (1 μg) was amplified under the following condition: pre-denaturation at 95°C for 5 min, 38 cycles of 95°C for 30 s, 62°C for 30 s, and 72°C for 30 s, followed by a final extension at 72°C for 10 min. The PCR product was further quantified by qPCR [22].

### Chromatin immunoprecipitation (ChIP)-qPCR

Binding relationship between *DNMT3B* and *miR-149* promoter in ESCC cells was determined by the ChIP assay as previously described [23]. When the cell confluence reached 80%, the cells were fixed in 1% formaldehyde at 23–25°C for 10 min and then ultra-sonicated into random chromatin fragments. The samples were centrifuged at 3,000 g at 4°C to collect supernatant, which was incubated with anti-DNMT3B (ab227883, 1:200, Abcam) or the control IgG (ab109489, 1:500, Abcam) at 4°C overnight. The endogenous DNA-proteinase complex was precipitated by Protein Agarose/Sepharose. After short centrifugation, the supernatant was discarded and the nonspecific binding was removed. The immunocomplexes were de-crosslinked at 65°C overnight, and the DNA fragments were extracted and purified by phenol/chloroform. The enrichment of miR-149 promoter fragments was examined by qPCR.

### Dual-luciferase reporter gene assay

The luciferase reporter vectors containing the wild-type (wt) 3ʹUTR of *RNF-2* (pGL3-RNF2-wt) or containing the mutant-type (mt) sequence between *RNF-2* and *miR-149* (pGL3-RNF2-mt) were provided by Promega Corporation, Madison, WI, USA. The renilla luciferase reporter vector pRL-TKs was used as positive control. The above vectors were transfected into HEK-293 T cells along with miR-149 mimic or NC mimic. After 24 h, the cells were lysed, and the luciferase activity in cells was examined by the dual-luciferase® reporter assay System (E1910, Promega). Relative luciferase activity was evaluated by the ratio of firefly luciferase activity to the renilla luciferase activity [24].

### Immunofluorescence staining

Cells on slides were fixed by pre-chilled acetone, washed by phosphate-buffered saline, and then incubated in 0.3% H_2_O_2_-supplemented 10% goat serum for 30 min. Thereafter, the cells were hybridized with anti-β-catenin (ab32572, 1:200, Abcam) overnight at 4°C, and then with HRP-conjugated secondary antibody (ZSGB-Bio Co., Ltd., Beijing, China) at 22–25°C for 45 min. The nuclei of cells were stained by 4’, 6-diamidino-2-phenylindole (Boster Biological Technology Co., Ltd., Wuhan, Hubei, China) [20].

### Western blot analysis

Cells were lysed in RIPA buffer (Beyotime) on ice for 5 min to collect total protein. After protein concentration examination by a bicinchoninic acid kit (Thermo Fisher Scientific), an equal amount of protein sample (30 µg) was run on 10% sodium dodecyl sulfate-polyacrylamide gel electrophoresis and loaded onto polyvinylidene fluoride membranes. The membranes were treated with nonfat milk at 20°C for 2 h and then hybridized with the primary antibodies (all diluted at 1:1,000) against Vimentin (ab22651, Abcam), β-catenin (ab231305, Abcam) and GAPDH (ab8245, Abcam) for 12 h at 4°C, and then hybridized with the secondary antibody (1:10,000, #7076, CST) for 1 h at 20°C. The protein bands were developed using the enhanced chemiluminescence kit (Thermo Fisher Scientific), and the protein quantification was conducted using an Image-Pro® Plus Software. GAPDH was used as the endogenous reference [25].

### Statistical analysis

Statistical analysis was conducted using SPSS21.0 (IBM Corp. Armonk, NY, USA). Normal distribution of all data was checked before the statistical analysis. Data were collected from three repetitions and expressed as the mean ± standard deviation. Differences between groups were analyzed by the *t* test, or by the one- or two-way analysis of variance. *p < 0.05 represents a statistical significance.

## Results

By using integrated bioinformatics analyses, we identified *miR-149* as an aberrantly downregulated miRNA in ESCC and *RNF2* as a candidate target while *DNMT3B* as an upstream epigenetic regulator of it. We hypothesized that *DNMT3B* possibly mediates *miR-149* promoter methylation to induce its downregulation and *RNF2* restoration, which therefore leads to ESCC progression. To validate this, the expression profiles of these molecules were detected in clinically collected tissues from patients with ESCC. ChIP-qPCR and luciferase assays were performed to validate the direct binding between these molecules. Altered expression of these molecules was induced in ESCC KYSE-150 and TE-1 cells to examine their functional interactions in the malignant behaviors of cells *in vitro* and tumorigenesis *in vivo*.

### miR-149 *is lowly expressed in ESCC tissues and cells*

**Figure 1.**
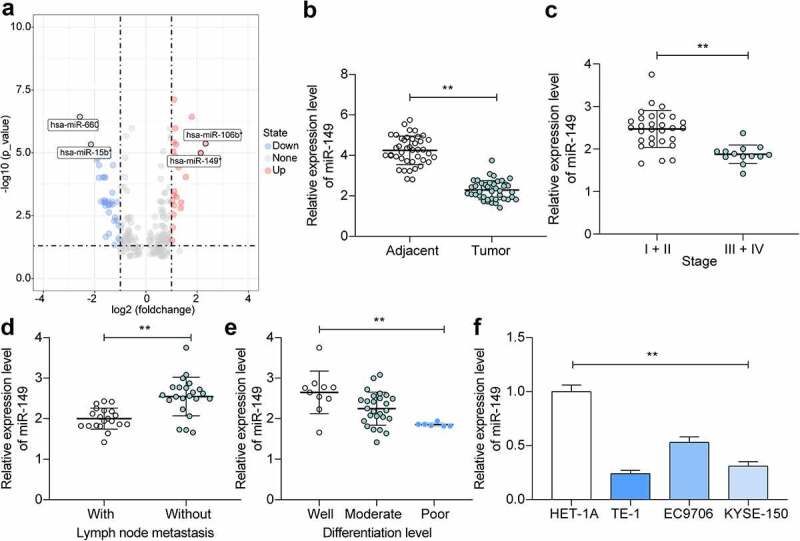
*miR-149* is expressed at low levels in ESCC tissues and cells. A, DE miRNAs between ESCC tumor and normal tissues screened using a GEO GSE67268 dataset; B, *miR-149* expression in ESCC and the paired adjacent tissue samples from 42 patients detected by RT-qPCR; C-E, association between *miR-149* and the clinical staging (c), lymph node metastasis (d) and tumor differentiation (e); F, *miR-149* expression in ESCC cell lines (TE-1, EC9706 and KYSE-150) and in HET-1A cells determined by RT-qPCR. In panels B-E, each plot indicates a single sample. **p < 0.01.

First, an ESCC-related miRNA expression dataset GSE67268, which comprises data from 113 cases of ESCC tumor tissue and normal esophageal tissue samples, was downloaded from GEO and analyzed. A total of 64 DE miRNAs were identified between normal and cancer tissues (Figure 1(a)). Among them, *miR-149* and *miR-106b* showed the greatest extend of decline in cancer tissues. While *miR-106b* has been largely investigated about its role in ESCC [26,27], less is known about the exact functions of *miR-149*. Therefore, we aimed to explore the function of *miR-149* in ESCC. Thereafter, the RT-qPCR detected reduced *miR-149* expression in tumor tissues compared to normal tissues (*p* < 0.0001) (Figure 1(b)). Furthermore, the link of *miR-149* to the clinical presentations of patients was measured. First, decreased *miR-149* expression was detected in the patients at advanced stages compared to those at early stages (*p* = 0.0002) (Figure 1(c)). Also, low *miR-149* expression was linked to positive lymph node metastasis (*p* < 0.0001) (Figure 1(d)) and poor tumor differentiation rate (*p* = 0.0017) (Figure 1(e)) in patients with ESCC. In cells, the *miR-149* expression level was lower in all ESCC cells than that in HET-1A cells (*p* < 0.0001) (Figure 1(f)). These results suggest that low *miR-149* expression is possibly linked to ESCC development.

### *Overexpression of* miR-149 *suppresses malignance of ESCC cells* in vitro

**Figure 2.**
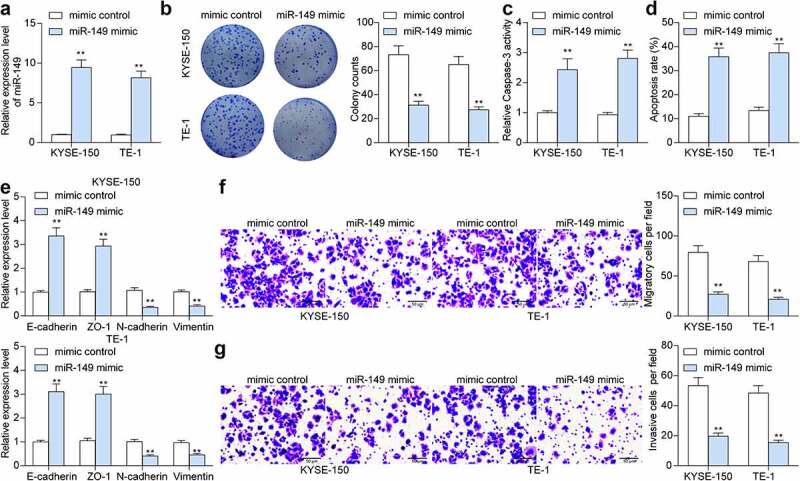
*miR-149* suppresses malignance of ESCC cells *in vitro*. a, expression of *miR-149* in KYSE-150 and TE-1 cells after miR-149 mimic or mimic control transfection detected by RT-qPCR; b, colony formation ability of cells; c, Caspase-3 activity in cells detected by a Caspase-3 kit; d, apoptosis rate in cells determined by flow cytometry; e, mRNA levels of the EMT-related factors (*E-cadherin, ZO-1, N-cadherin and Vimentin*) in ESCC cells measured by RT-qPCR; f-g, migration (f) and invasiveness (g) of cells evaluated by Transwell assays. **p < 0.01.

To verify the functions of *miR-149* in ESCC cell development, overexpression of *miR-149* was induced by transfecting miR-149 mimic into KYSE-150 and TE-1 cells, and the transfection efficiency was examined by RT-qPCR (*p* < 0.0001) (Figure 2(a)). The upregulation of *miR-149* decreased the counts of colonies formed by ESCC cells (*p* < 0.0001) (Figure 2(b)). Apoptosis of cells was further determined by the activity of pro-apoptotic Caspase-3 in cells and the flow cytometry. miR-149 mimic significantly increased Caspase-3 activity in cells (*p* < 0.0001) (Figure 2(c)), and the flow cytometry directly demonstrated that the cell apoptosis rate was elevated after *miR-149* upregulation (*p* < 0.0001) (Figure 2(d)). Further, the epithelial–mesenchymal transition (EMT) activity in cells was measured. The RT-qPCR results showed that the mRNA levels of epithelial markers *E-cadherin* and tight junction protein 1 (*ZO-1*) were increased, whereas the mRNA levels of mesenchymal markers *N-cadherin* and *Vimentin* were decreased after *miR-149* upregulation (*p* < 0.0001) (Figure 2(e)), indicating that *miR-149* diminishes EMT activity of ESCC cells. Moreover, according to the Transwell assay, the migration and invasiveness of KYSE-150 and TE-1 cells were declined after miR-149 mimic transfection (*p* < 0.0001) (Figure 2(f,g)).

### miR-149 *suppresses growth of ESCC cells* in vivo

**Figure 3.**
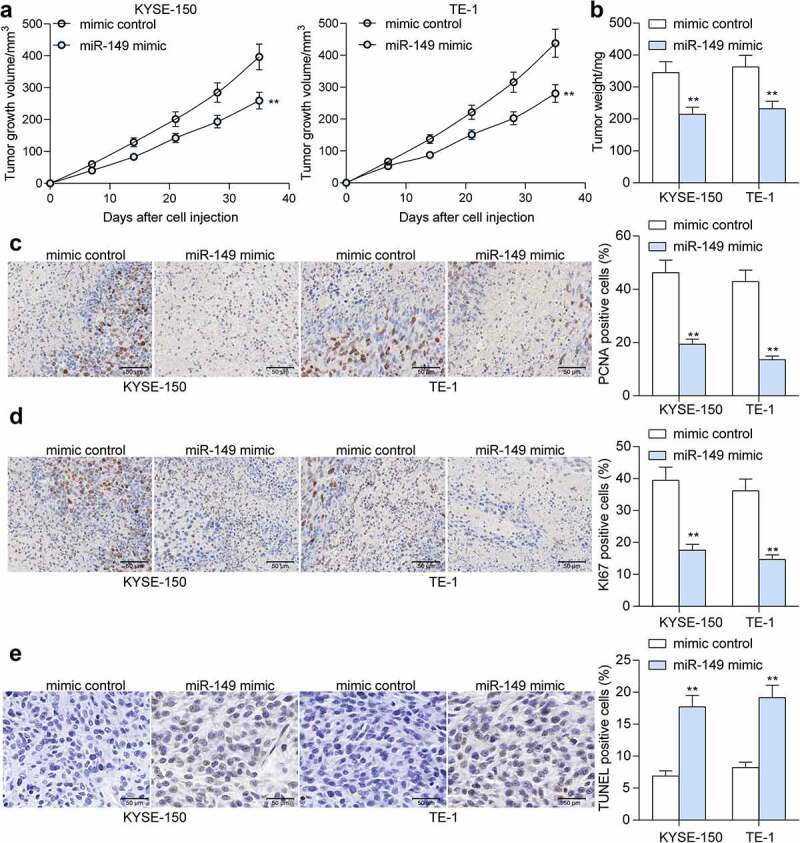
*miR-149* suppresses growth of ESCC cells *in vivo*. a, weekly change in the volume of xenograft tumors; b, tumor weight on the 36^th^ day; c-d, expression of PCNA (c) and KI67 (d) in tumor examined by IHC; E, cell apoptosis rate in tumor tissues determined by TUNEL assay. N = 6 in each group. **p < 0.01.

To validate the function of *miR-149* in ESCC cell growth, cells stably transfected with mimic control or miR-149 mimic were implanted into NOD/SCID mice. After that, the volume of xenograft tumors was determined every 7 days. *miR-149* upregulation in cells slowed down the tumor growth *in vivo* (*p* < 0.0001) (Figure 3(a)). On the 36^th^ day, the animals were sacrificed to collect xenograft tumors. Also, the tumor weight was decreased when *miR-149* was upregulated (*p* < 0.0001) (Figure 3(b)). In addition, the IHC of tissue sections suggested that the levels of KI67 and PCNA (tumor proliferation markers) in cells was declined by miR-149 mimic (*p* < 0.0001) (Figure 3(c,d)), and the portion of TUNEL-positive cells in tissues was increased (*p* < 0.0001) (Figure 3(e)).

### The promoter region of miR-149 is highly methylated in ESCC

**Figure 4.**
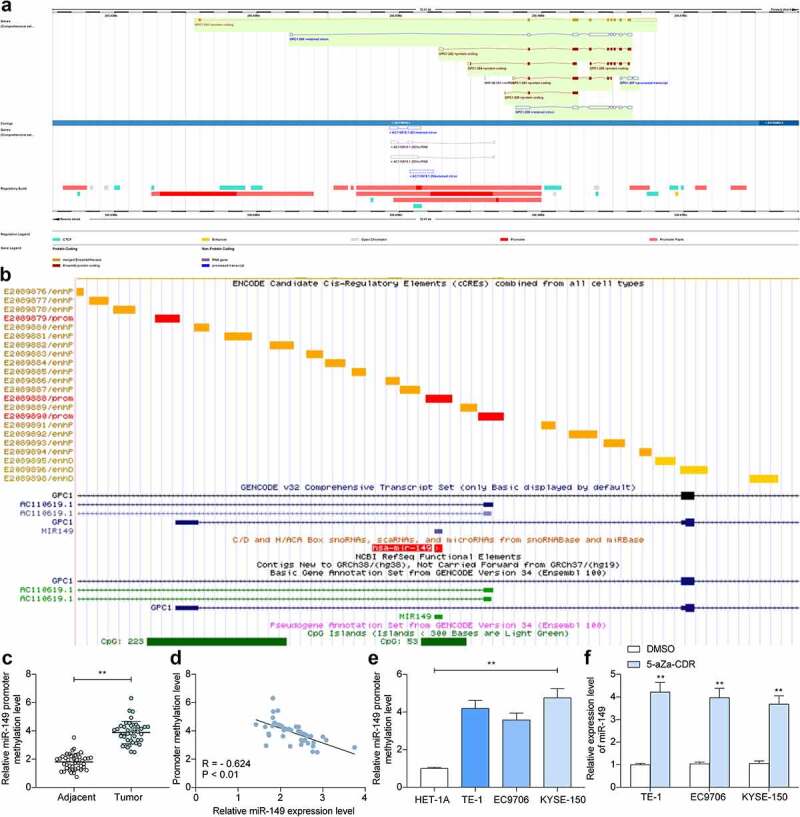
The promoter region of *miR-149* is highly methylated in ESCC. a, location of *miR-149* and *GPC1* in genome measured using the Ensembl system; b, the CpG Island on the miR-139 promoter predicted using the UCSC browser; c, promoter methylation level of *miR-149* in tumor and healthy tissues examined by MSP-qPCR; d, correlation between the promoter methylation level of *miR-149* and its expression in tumor tissues; e, promoter methylation level of *miR-149* in ESCC cell lines (TE-1, EC9706 and KYSE-150) and in HET-1A cells determined by MSP-qPCR; f, *miR-149* expression in TE-1, EC9706 and KYSE-150 cells after 5-aZa-CDR treatment determined by RT-qPCR. In panels C and D, each spot indicates a single sample. **p < 0.01.

We then focused on the potential upstream regulators of miR-149. According to the bioinformatics analysis on the Ensembl system (http://www.ensembl.org/index.html), *miR-149* and glypican gene (*GPC1*) were suggested to belong to a common transcript (Figure 4(a)). We then observed a C-phosphate-G (CpG) Island on the *miR-149* promoter sequence according to the data in UCSC (https://genome.ucsc.edu/index.html) (Figure 4(b)). We then wondered whether the *miR-149* promoter is methylated in ESCC cells, which consequently leads to *miR-149* downregulation. To validate this, we determined methylation of the *miR-149* promoter in 42 ESCC patients through the MSP-qPCR assay, which showed the promoter methylation of *miR-149* was enhanced in the tumor tissues (*p* < 0.0001) (Figure 4(c)). In addition, in the collected 42 pairs of ESCC tumor samples, the methylation level of *miR-149* was inversely linked to its expression level (*p* < 0.0001) (Figure 4(d)). Moreover, *miR-149* promoter methylation was greater in ESCC cells than in HET-1A cells (*p* < 0.0001) (Figure 4e). The ESCC cells were further treated with 5-aZa-CDR, a DNMT inhibitor, after which the *miR-149* expression was significantly elevated (*p* < 0.0001) (Figure 4(f)).

### DNMT3B *regulates methylation of* miR-149 *promoter*

**Figure 5.**
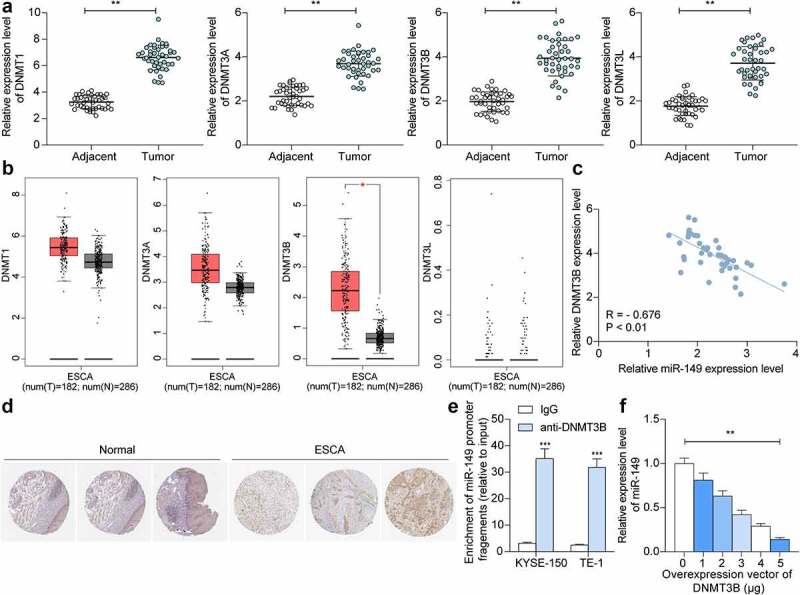
*DNMT3B* regulates methylation of *miR-149* promoter. a, *DNMT1, DNMT3A, DNMT3B* and *DNMT3L* mRNA levels in ESCC and normal tissues from 42 collected patients detected by RT-qPCR; b, *DNMT1, DNMT3A, DNMT3B* and *DNMT3L* expression predicted in TCGA-ESCA; c, an inverse correlation of *miR-149* and *DNMT3B* in ESCC tissues; d, strong positive staining of DNMT3B in ESCC; E-F, binding between DNMT3B and miR-149 promoter verified through ChIP-qPCR (e) and luciferase (f) assays. In panels a and c, each spot indicates a sample. **p < 0.01.

The above findings suggested that methylation of the *miR-149* promoter might reduce *miR-149* transcription and expression, therefore promoting growth and dissemination of ESCC cells. To explore the regulators responsible for this event, we focused on four common methyltransferases *DNMT1, DNMT3A, DNMT3B,* and *DNMT3L*. First, we determined expression of these four candidate genes in the collected tissues. It was found that all of them were highly expressed in ESCC tissues (*p* < 0.0001) (Figure 5(a)). Thereafter, we further searched the expression profiles of *DNMT1, DNMT3A, DNMT3B* and *DNMT3L* in TCGA-ESCA. It was suggested that they were upregulated in the cancer tissues, though, only *DNMT3B* expression showed a significant difference (Figure 5(b)). Thereafter, we determined the relevance of *miR-149* to *DNMT3B* expression in the tumor tissues. The *miR-149* expression showed an inverse correlation with *DNMT3B* expression in 42 ESCC patients (Figure 5(c)). In addition, the IHC assay suggested that *DNMT3B* showed strong positive staining in ESCC tissues (Figure 5(d)). To confirm the binding of *DNMT3B* with *miR-149*, a ChIP-qPCR assay was conducted, in which an enrichment of *miR-149* promoter fragments was detected in the complexes reacted with anti-DNMT3B (*p* < 0.0001) (Figure 5(e)). Also, the luciferase assay suggested that co-transfection of pGL3-Enhancer luciferase vector containing the *miR-149* promoter sequence and overexpression vector of *DNMT3B* led to a decreased luciferase activity in 293 T cells (*p* < 0.0001) (Figure 5(f)). These results indicated that *DNMT3B* can bind to the *miR-149* promoter and suppress its expression.

### miR-149 *directly targets* RNF2 *mRNA*

**Figure 6.**
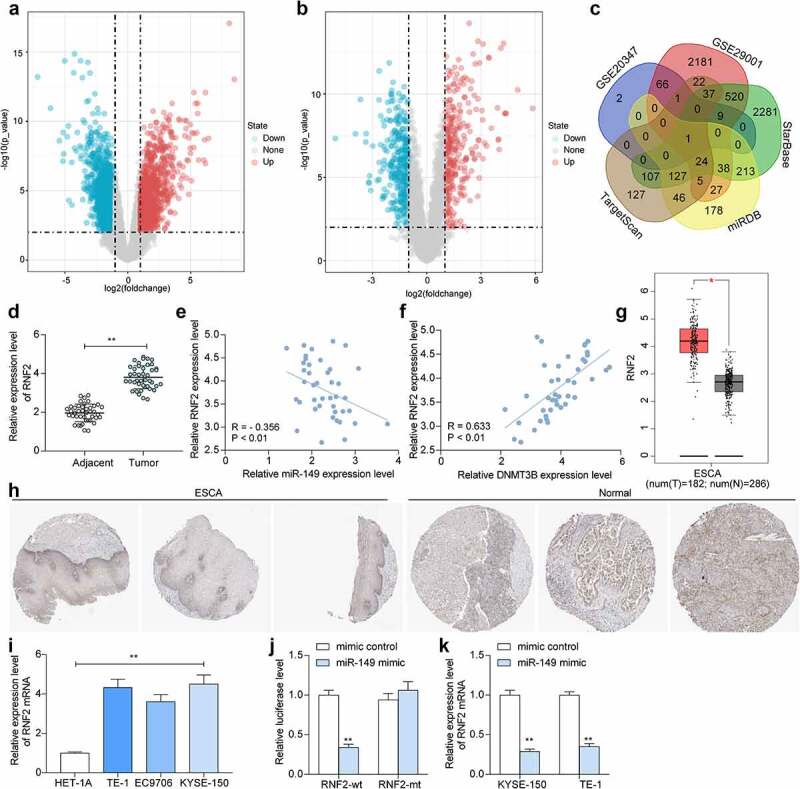
*miR-149* directly targets *RNF2* mRNA. a-b, DE genes between ESCC and normal tissues analyzed using the GEO GSE29001 and GSE20347 datasets; c, a Venn diagram for target mRNAs of *miR-149* predicted via bioinformatics systems and DE genes screened from two datasets; d, *RNF2* mRNA in ESCC and normal tissues detected by RT-qPCR; E-F, correlation of *RNF2* expression with *miR-149* (e) and *DNMT3B* (f) in ESCC tumor tissues; g, predicted *RNF2* expression in TCGA-ESCA; H, staining intensity of RNF2 in ESCC and normal tissues examined by the IHC assay; i, mRNA expression of *RNF2* in ESCC cells and in HET-1A cells determined by RT-qPCR; j, binding between *miR-149* and *RNF2* verified by luciferase assay; k, *RNF2* mRNA expression in TE-1 and KYSE-150 cells after miR-149 mimic transfection determined by RT-qPCR. In panel D-F, each spot indicates a sample. **p < 0.01.

We next focused on the downstream molecules modulated by *miR-149*. The GEO GSE29001 dataset containing data of 21 ESCC tumor tissues and 24 healthy tissues, and another GSE20347 dataset containing data of 17 tumor tissues and 24 healthy tissues, were analyzed to screen the DE genes. A total of 163 and 241 DE genes were screened, respectively (Figure 6(a,b)). After that, the potential target transcripts of *miR-149* were explored on three bioinformatics systems: StarBase (http://starbase.sysu.edu.cn/), TargetScan (http://www.targetscan.org/vert_72/), and miRDB (http://mirdb.org/). The outcomes were compared to the upregulated mRNAs screened above, and *RNF2* was identified (Figure 6(c)). Thereafter, we explored *RNF2* expression in ESCC. The RT-qPCR result suggested that the *RNF2* expression was increased in the tumor tissues compared to the normal tissues (*p* < 0.0001) (Figure 6(d)), which presented an inverse association with *miR-149* while a positive association with *DNMT3B* (*p* < 0.0001) (Figure 6(e,f)). Similar trends were obtained from TCGA-ESCA and the IHC assay results (Figure 6(g,h)). Moreover, the *RNF2* expression in cells was determined. Again, high *RNF2* expression was detected in the ESCC cell lines compared to the HET-1A cells (*p* < 0.0001) (Figure 6(i)). The binding relationship between *miR-149* and *RNF2* was validated. miR-149 mimic significantly reduced the luciferase activity of pGL3-RNF2-wt plasmids in 293 T cells (*p* < 0.0001) (Figure 6(j)). Moreover, upregulation of *miR-149* decreased *RNF2* expression in KYSE-150 and TE-1 cells (*p* < 0.0001) (Figure 6(k)), indicating that *miR-149* directly targets *RNF2* mRNA.

### *Overexpression of* RNF2 *enhances growth and metastasis of ESCC cells*

**Figure 7.**
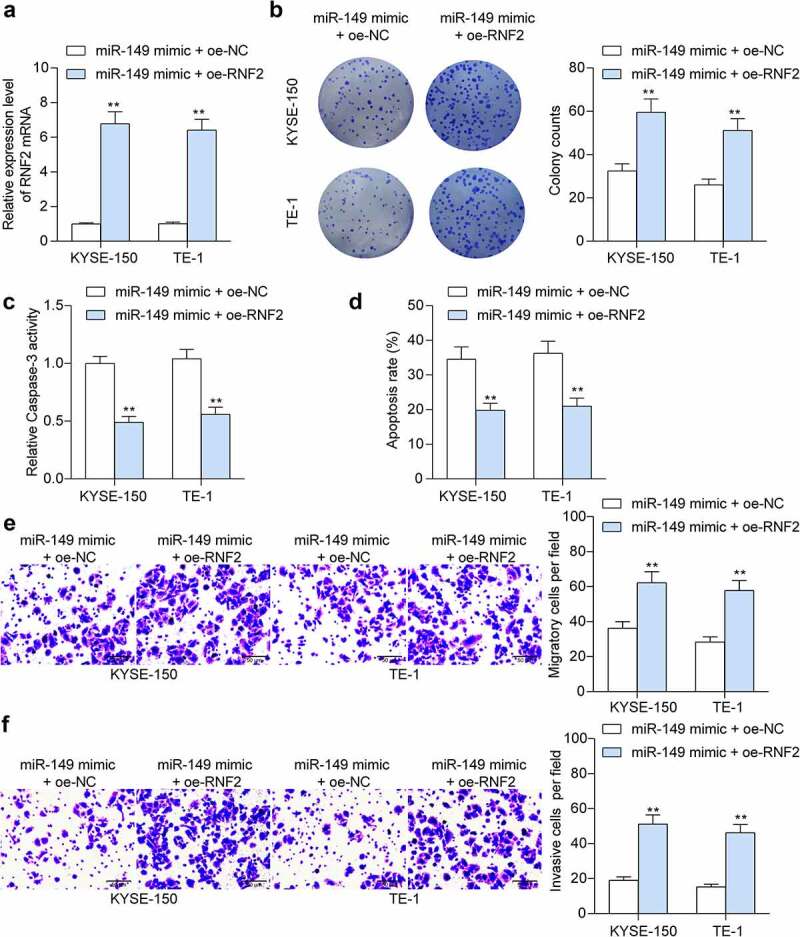
Overexpression of *RNF2* promotes ESCC cell growth and metastasis. a, *RNF2* mRNA in KYSE-150 and TE-1 cells after oe-RNF-2 administration examined by RT-qPCR; b, colony formation ability of cells; c, Caspase-3 activity in cells determined using a Caspase-3 kit; d, apoptosis of cells detected by flow cytometry; E-F, migration (e), and invasiveness (f) of cells measured by Transwell assays. **p < 0.01.

To further explore if *miR-149* inhibits *RNF2* to suppress ESCC cell growth, upregulation of *RNF2* was administrated into KYSE-150 and TE-1 cells overexpressing *miR-149*. The successful transfection was detected by RT-qPCR (*p* < 0.0001) (Figure 7(a)). Thereafter, the proliferation of KYSE-150 and TE-1 cells blocked by miR-149 mimic was restored following *RNF2* overexpression (*p* = 0.0001) (Figure 7(b)). In addition, the Caspase-3 activity and cell apoptosis rate were decreased by *RNF2* (*p* < 0.0001) (Figure 7(c,d)). The metastatic potential of cells was determined as well. The reduced migration and invasiveness of KYSE-150 and TE-1 cells were enhanced after *RNF2* upregulation (*p* < 0.0001) (Figure 7(e,f)). This body of evidence validated that downregulation of *RNF2* is accountable for the anti-cancer effects of *miR-149*.

### RNF2 *activates the Wnt/β-catenin signaling to promote ESCC progression*

**Figure 8.**
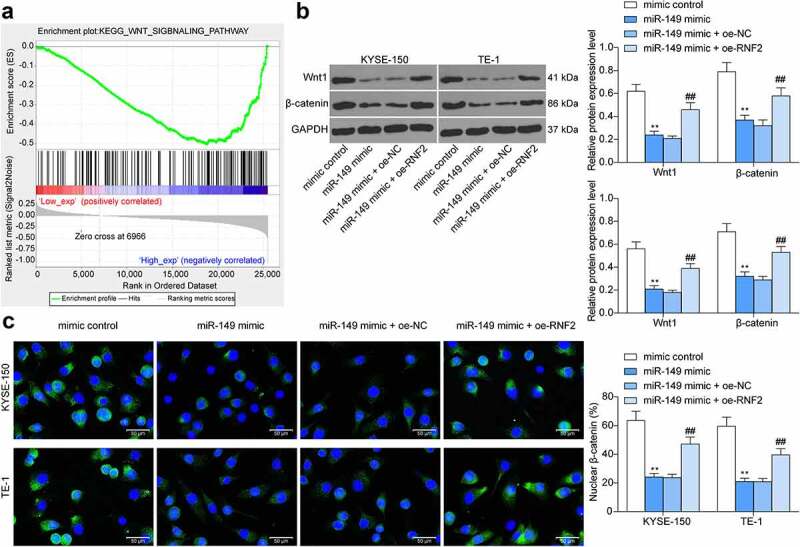
*RNF2* activates the Wnt/β-catenin pathway. a, the *RNF2*-related signaling pathways predicted using GSEA; b, protein levels of Wnt1 and β-catenin in ESCC cells detected by Western blot analysis; c, sub-cellular localization of β-catenin in cells examined by immunofluorescence staining. **p < 0.01.

The Gene Set Enrichment Analysis (GSEA) was performed to explore the RNF2-related pathways, which suggested that high expression of RNF2 was positively linked to the activity of the Wnt/β-catenin pathway (Figure 8(a)). Therefore, the Wnt/β-catenin activity in ESCC cells was determined. It was observed that the Wnt1 and β-catenin protein in ESCC cells were decreased by miR-149 mimic but then restored after *RNF2* overexpression (*p* < 0.0001) (Figure 8(b)). Immunofluorescence staining further validated that the β-catenin nuclear translocation in cells was decreased by miR-149 mimic but increased by oe-RNF2 (*p* < 0.0001) (Figure 8(c)).

## Discussion

Despite the advances in the therapeutic options including adjuvant chemotherapy and radiotherapy, ESCC remains a huge challenge due to its aggressive pathological features and poor prognosis [4,28,29]. Epigenetic and genomic changes are frequently implicated in cancer initiation and development. In this work, we report that *DNMT3B* suppresses *miR-149* by regulating DNA methylation, which enhances *RNF2* expression and activates the Wnt/β-catenin pathway, thus triggering the growth and metastasis of ESCC cells.

The GEO datasets containing gene expression data are helpful tools in the screening of abnormal gene expression profiles in specific pathological conditions [30]. In this study, the bioinformatic analysis using GSE67268 dataset predicted a low-expression profile of *miR-149* in ESCC tumor samples. Reduced expression level of *miR-149* was then detected in tumor tissues and ESCC cells. The tumor-inhibiting functions of *miR-149* have been well established. For instance, *miR-149* was documented to inhibit metastasis of breast cancer by abrogating the paracrine interactions with macrophages, and poor expression of *miR-149* was linked to reduced patient survival [31]. Likewise, restoration of miR-149 has recently reported to delay tumorigenesis of breast cancer [32]. This miRNA was also reported to inhibit the growth and aggressiveness of human lung cancer through a FOXM1/cyclin D1/MMP2 axis [33]. Here, we observed that low *miR-149* expression was also linked to increased lymph node metastasis and poor survival rate in patients. Further upregulation of *miR-149* suppressed growth, metastasis, whereas augmented apoptosis of ESCC cell lines both *in vitro* and in nude mice. This was quite in agreement with the report by Xu *et al*. that artificial upregulation of *miR-149* impeded proliferation and aggressiveness of ESCC KYSE-450 cells [9].

On the basis of this finding, we further investigated the molecules involved. Bioinformatics analyses using the Ensembl and UCSC browsers suggested there is a CpG Island on the *miR-149* promoter, and then a high level of *miR-149* promoter methylation was found in ESCC tumors and cells. Among several DNMTs, *DNMT3B* was abundantly expressed in tumor tissues and directly bound to *miR-149*. DNA methylation has been recognized as epigenetic therapeutic targets in cancer management [34]. Abnormal activity of DNMTs and dysregulation of DNA methylation are closely correlated with multiple forms of tumor. The high-expression profile of *DNMT3B* has been found in several studies. The incidence of nuclear immunoreactivity of DNMT3B was significantly increased in esophageal cancer samples compared to the paired nonmalignant epithelium, which was relevant to distant metastasis as well [35]. Similar trends were observed in a recent report by Su *et al*., which suggested that *DNMT3B* and the aberrant DNA methylation activity played oncogenic roles in ESCC [36]. In melanoma, *DNMT3B* was found as a negative regulator of *miR-196b*, and *DNMT3B* loss reduced *miR-196b* promoter methylation and increased its expression, which suppressed formation and growth of melanoma [37]. Similarly, high expression of *DNMT3B* and *DNMT3A* suppressed expression of *miR-29* through methylation modification and induced disease progression in Burkitt lymphoma [38]. In this paper, we found the DNMT inhibitor 5-aZa-CDR led to increased *miR-149* expression, indicating that poor expression of *miR-149* was at least partially, regulated by *DNMT3B*.

The subsequent bioinformatics analyses using three predicting websites and two GEO datasets suggested *RNF2* as a target transcript of *miR-149. RNF2* is a common oncogene in human malignancies whose downregulation blocked cell proliferation whereas increased cell cycle arrest and apoptosis [39–41]. Interestingly, a similar miR-149-RNF2 axis has been found in gastric cancer [42]. Rather than limiting in confirming the binding relationship between *miR-149* and *RNF2*, our present study found that the malignant activity of ESCC cells suppressed by *miR-149* was recovered after further *RNF2* overexpression. In addition, we further explored that *RNF2* expression was possibly correlated with the activity of the Wnt/β-catenin pathway in ESCC. Wnt/β-catenin is one of the most common signaling pathways activated [43]. This is also applied in ESCC, while suppression of this pathway has been well established to reduce the malignant behaviors of ESCC cells [44]. The activity of Wnt/β-catenin, in the present research, was initially blocked by miR-149 mimic but then recovered upon *RNF2* overexpression, indicating that the RNF2-Wnt/β-catenin cascade was accountable for the development of ESCC after miR-149 downregulation.

## Conclusion

**Figure 9.**
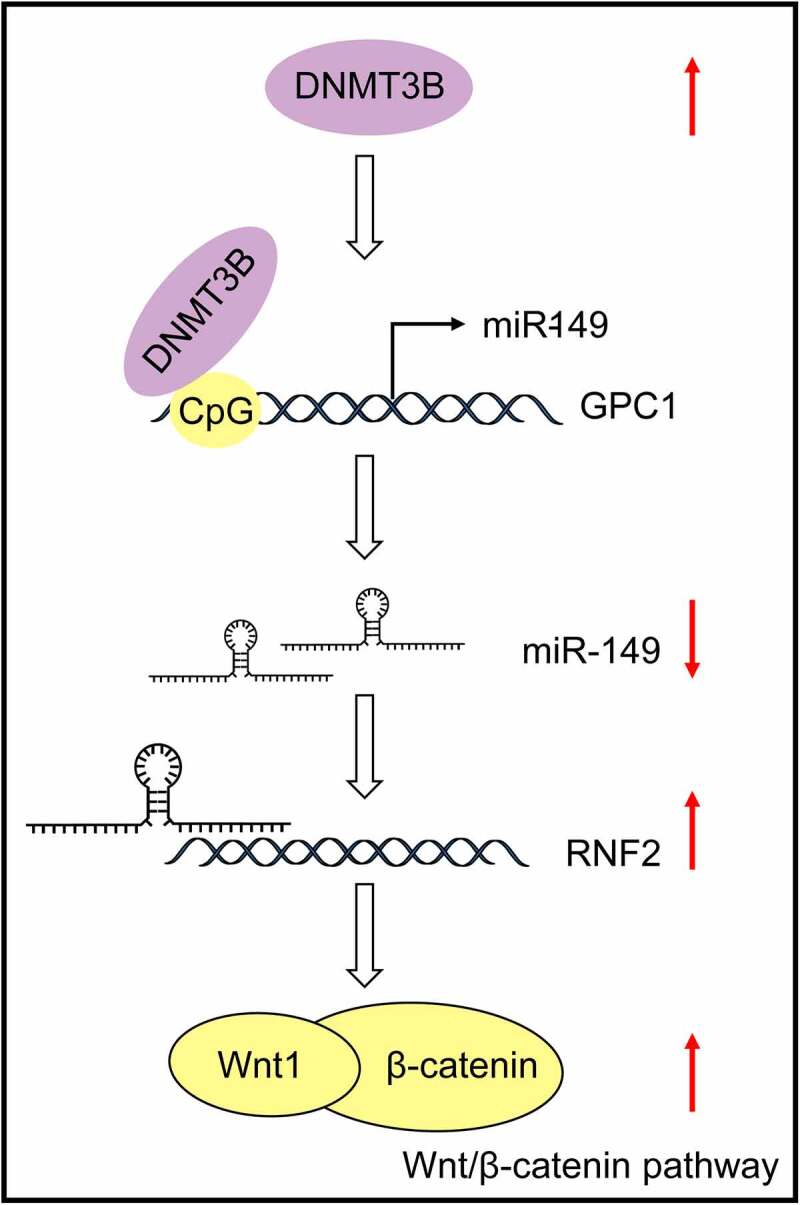
A graphic abstract. *DNMT3B* induces CpG Island methylation at the promoter of *miR-149* on the *GPC1* transcript, which results in *miR-149* downregulation and restoration of *RNF2* mRNA, leading to Wnt/β-catenin pathway activation and augmented development of ESCC.

In summary, this study demonstrates that highly expressed *DNMT3B* in ESCC promotes *miR-149* promoter methylation to reduce its expression. Downregulation of *miR-149* led to increased *RNF2* expression and Wnt/β-catenin activation, thus triggering growth and metastasis of ESCC cells (Figure 9). However, there might be more upstream regulators responsible for *miR-149* downregulation and downstream effectors of *miR-149* involved in tumor progression. Moreover, the exact mechanism by which *RNF2* regulates the Wnt/β-catenin signaling remains unknown yet. The potential clinical diagnostic and prognostic values of *miR-149* also demand validation via follow-up studies and more pre-clinical researches. We would like to focus on these issues in our further studies. Anyway, we hope these findings may offer new understandings in the onset and progression of ESCC, which may be helpful in developing novel therapeutic strategies for ESCC.

## Data Availability

All the data generated or analyzed during this study are included in this published article.
